# Record of Meteorological Conditions at Buffalo, for November, 1854

**Published:** 1855-01

**Authors:** Sanford B. Hunt


					﻿ART. X.—Record of Meteorological Conditions at Buffalo, for November,
1854. By Sanford B. Hunt, M. D.
■a.injH.iadumx, 01 2 ~	05	0310 1/5 1/3 50 2 ? *T +1S "	1-1	00	! 0	~
JO oSuBJ JSOJBOJr)	X	++ S*
■Xu I m o co i' c e: 1- i.-j c tt 71 x t-. ju Ti o“inrm n k> is c n m n'co o o ' '
nrmnTT rmirj	7! -H r' /l 71 O l7 J. CO O O', 17 X ® 17. 0 C7 - I' >7 t - *f r. -cf
-pitunH uBSWI oc t-t-aooot-oooooot-ap r-_op oooooomooaommmmtTooooooooooao oo
. (h	T m h<o co rf-fd i^n h dco a mt'n r-o o m d m naicio in m 71 -i
P1!0,! C: h co t: -7	71 <7 io 71 o -■ h	ir, « 7 X ® 71 - 7 7 - th
*	5	A'0(I utsaiAi idai^md oocod o’cosixi oxidrirfo hh	oio odd ui
g _____________CO	CO d d CO CO COCOTTn'COCOCOCOCO CO-00-70 CO d CO -? n< CO d co dco d_cn_
■am™ iCOCO COCO COCO COCO	CO CO	COCOCOCOCO	COCOCO	CO|TJ<
JJUIB CO CO . CO CO . co co .coco . .co .co . co co co co co cococo . co m
-jadraax ubojuL-i cri d’ in co co d ci o d oo t-i o’ t~ m co’t'P d cd in cd d —' rPcd ci m
_____________I in -h« co co tT m< co m -th coco co co coco coco co th ,n th co co co co dco_
.	oinoooomoooooinoinoinommininino m m m o o o i co
. 'AjiptUinH COOOrHCO—<CO—iCOCO-t—(COCOCOCOCOCOCOCOCOCOCOCOCOCOCOCOCOi—ICOiO CO
'	£ _______aocoooaoooooooooooooaooomoomoomoooi mmmmoomooco cooo oo I oo
g	coco co co coco cocococo cococo co co cocococo coco co co co co’-f
8 •1TITOJ Marr	•C0.	•C'?	. CO .CO. COCOCOCOCOCOCOCOCOCOCOCOCOCOCOCO co co -r
o *u.ud ■—i th oo r-i oo t"’ d m’ co in o m’ th —< e-i ci in .-i o’ ci •-< nr co’ o’ oo ci r-i co co th
r	o o c: d71 c: co co co-r O CC CO CC 71 n CO ® 7tCO7O O -f CO717! 7!C0 ci co
si	............................................ =r
.	a.mjtauwj, cD’coasCTtoCTo^oGdCTtnoi^cDo^GdcDcoCT'^r^c^tri^^CTi^GO, go &>
-H __________T!<kQeQCQCQxt«C0^0QmT^u^gpCQT^COCQCOCQCQCQCQ^Tj<^CQCQ cc CG CT CO_
to h h io w 4 o m	h- j	h oi q d o co to <o e6 oi _____
spnoo JO JtUI v|i"OOOO^OCO'Tj’OOOOOOOOOOOOOOOOOOGOOC7iOi
7	tomoootoootQooto o to o ©io o o »o o tn o to o o o o o »n i —<
. AlipiUlnH
£____2____h oo »n r- ^0 oo o co t— o ao_co Ci oo cc co go oo go qo oo cd I r—
■£	70 CO CO CO CO	CO CO CO CO	CO CO CO CO CO CD CO CD CO CO	CO CO I o
c .nnTnj M _. co co co co co . . co co co co . . co co co . co -co <d co cd co co . . .co . co It-
§ 4uI°d AV,a —> cn qq co rH ct cd co ao co o co co co co o —< o co —- ai cd go o aS -J c$ |co
. u	-H CO CT CT co co co co co co co CO CO O'? co co co co CO co CO CT co CT CO CT I co
£-----------------------------------—	■	g—
•	. -oin^auidj, coCTCTCTJoor^aSCTco’tnaScioCTQOc^t^odtO'^iocDCD^cotHt-^t'aotD ct
q pL| _________in UQ ^^CO	M? Td? AQ*^ ;TF CO CO CO fp.CQ.OOCQ CO CD CO CO CO CO CO __
!	o o'	. J3 ~
“	=s§®£ l^^^’^WajoQR^aioQajajoQaicQ^ccaiaj ajodoo^S^FF
Pi	.......................... ..«	.	.	•	. »
g	_________|Tft Tfl C^^co —»	>"< rH CO co in tn r-< G9 CO GM CM >-< O O CT »-< »o w c* __
p | SpnOjQJO^	T“^	GO CO <O ^0 OJ <D CO (OJ ^0 ^0 ^0
»n co o o o co o co o co	»n co o o	tn	co	tn	o	co	»n in	tn	o o tn o o m
. rnTTATTTTn r, CO '-D —I *O CD—<CDC0 *D> CD	CO CD CD CD	CO	CD	CO	CD	O	CO CO	CO	—< CO CO CD CD CO	00
A-iIPJuinH ^QoaoooaocoGOGOQOc—c^ooaooo	crj	'X	0	go	o	o a;	0	oo7?cnooooc^	oo
© __________________________.	_______________________ **"*_______i___.
O	CD CO COCO COCOCOCOCDCOCOCO CD CO CO CO CO CO CD	CO CD co co co co
g.	.coco . CO CO CO CO. CD CO CO CO CO. CO CD CO .CDCOCD . co co. co. co CO CT
•	o	’?uI°d M9U CTODacoCTcdcoCT’^oai'^Gdo	cd	—n o ct o »n	coo^cor-cd cd
%_________in CO CO CO CT co co co co-yco co co	co	co co CO CO CT	CO CO	CO CO CO CT CT CT co
•	m	r°
<1 -ajnj.duiax p,- lfj in t-i o 07 o -i ni rd >n cd cd in d cd d< oo cd in -d d cd t-i
«	m n1 ■* co 7i-Ti n* cn co n n co-f cn_co oo co co cn d co co _ - n n x n 71 co
•<	Sts £ iJs’sd • •	PQ	o o . .	fe-u-u-i
o§2®£	m «2 aj f?
<3 £ ............................ . . .rjC. . . . .
co d co i—1 ocococoi-<i-<i-ii-<co-^r-idin'-ii-<coooi-id-^cod co co 1
spnopjoj.arv oom o o c~ o o o o o o m o o o o o o o o m m 60 o Q-gm |
o' ui 2	S ''ij <2	5
•papaantBj[ 1< Jj	.SP	.2PS P-i.5Ps P-i.SP«	S	.£®
m	CT
_	O *»___________________________________—___________________________________
is s	s	Ms
-treSaa ureg	Ph	Ph	Ph Ph <j <1 Ph	<i .SP	<< <<
g	|ao	co	t- omoodjz; ao^;	o co
®	th	in	m i-H rH t- o th tj< o	i—< o in	o^d
n<	co	in oo x- m co m co m	-m< m d	md	co
■a™}! jo ‘al	o	Ti*	d d. o. n< o co r-	d d	d	o
*_________'...............TH —i	________‘ _____________r—‘
jOOCOLO	00	COOOOOO. CdOCnrH	OOd. dm
•uioJBg jo ’uj • m-	or;	oo oo'm m m‘ci ci m m oo oo	ci ci m ci oo
171 71 7! 71	d______________d d d d d dddddd	dddd d_
-------	Tth d co rrincot^oomoi-Hdco-mincot^ oo m oh dccom^i-xmo
•OJBgi________________rHi-HTHi-Hi-HTHrHrH-HTHddddddddddCO__________________________
Snow in evening, f Gale from S. W. at 10, A. M. with snow, j: Drizzly day. § Cloudy; clear at
night. || Snow squall at 7, A. M. and 9, A. M.; heavy rain in P. M. Snowed all day; heavy snow at
4, P. M. *• About 9 inches of snow on the ground, jf Snow from 8, A. M. till noon, ft Showers.
§iSnow during night.
				

